# Hypercomplex extreme learning machine with its application in multispectral palmprint recognition

**DOI:** 10.1371/journal.pone.0209083

**Published:** 2019-04-15

**Authors:** Longbin Lu, Xinman Zhang, Xuebin Xu

**Affiliations:** 1 MOE Key Lab for Intelligent Networks and Network Security, School of Electronics and Information Engineering, Xi’an Jiaotong University, Xi’an, Shaanxi, China; 2 Guangdong Xi'an Jiaotong University Academy, Foshan, Guangdong, China; The University of Memphis, UNITED STATES

## Abstract

An extreme learning machine (ELM) is a novel training method for single-hidden layer feedforward neural networks (SLFNs) in which the hidden nodes are randomly assigned and fixed without iterative tuning. ELMs have earned widespread global interest due to their fast learning speed, satisfactory generalization ability and ease of implementation. In this paper, we extend this theory to hypercomplex space and attempt to simultaneously consider multisource information using a hypercomplex representation. To illustrate the performance of the proposed hypercomplex extreme learning machine (HELM), we have applied this scheme to the task of multispectral palmprint recognition. Images from different spectral bands are utilized to construct the hypercomplex space. Extensive experiments conducted on the PolyU and CASIA multispectral databases demonstrate that the HELM scheme can achieve competitive results. The source code together with datasets involved in this paper can be available for free download at https://figshare.com/s/01aef7d48840afab9d6d.

## 1 Introduction

Nowadays, machine learning has been playing an increasingly significant role in our daily life and a variety of machine learning areas have attracted great interests from researchers. The neural network technology, as a kind of typical machine learning method, is proven to be a successful tool for artificial intelligence (AI). With the rapid development of computer hardware, deep neural network techniques also achieve a huge success in various kinds of recognition tasks [1, 2].

Recently, a novel machine learning theory called extreme learning machine (ELM) was proposed by Huang et al. [3, 4] and has aroused growing worldwide concerns. It is a machine learning method for single-hidden layer feedforward neural networks (SLFNs) that differs from the traditional back-propagation (BP) algorithm and its variants [5–7]. Unlike the BP-based learning approach, which employs an iterative process to tune the hidden nodes of an SLFN, the ELM method completes a training task without any repeated optimizing steps. An ELM randomly assigns the input weights and bias of an SLFN and analytically calculates the output weights by a simple Moore–Penrose generalized inverse. This algorithm can avoid many difficulties of conventional learning methods, such as the settings of stopping criteria, learning rates and learning epochs. ELM has been shown to be able to find a global optimal solution with excellent universal approximation ability and a very fast learning speed. The advantages of ELM—computation cost and generalization performance—render it one of the most popular machine learning methods, with extensive and successful applications in classification, regression, clustering, compression and feature learning problems [8–12].

In the past decade, a variety of ELM variants were proposed to address problems in the original theory; they have significantly enhanced the contributions of ELM to theoretical studies and engineering applications. For example, a regularized extreme learning machine [13] was investigated to solve the overfitting problem based on structural risk minimization principle and weighted least squares. In [14], a fast and accurate online sequential learning variant was developed and applied to gesture recognition and object tracking; it has shown excellent performance with regard to accuracy and computation time. In [15], the original ELM model was extended to complete classification and regression tasks with noisy or missing data. From the implementation aspect, a stacked ELM invariant [16] was designed to render it feasible for large data sets and real-time reasoning. In recent years, the ELM concept has been introduced in multilayer perception [17, 18]. As demonstrated in [17], compared with the greedy layer-wise training of deep learning, the ELM-based framework has a substantially better learning efficiency.

Although ELM has been successfully applied in an extensive range of domains, it is primarily utilized for single-source information classification. In the case of multisource features, a fusion operation must be performed either at the feature level or the matching score level [19–21] to achieve a final result. For feature-level fusion, multisource features are simply jointed. The input hidden nodes of ELM are adjusted to the dimension of the joint features, which generates a very large computational cost. Regarding matching score level fusion, features from each channel are calculated to separately obtain a matching score by ELM; these scores are fused for the final decision. This strategy merely considers the matching score information that has lost some discriminative features. Therefore, the high accuracy of the fusion at the feature level cannot be achieved. In this paper, we propose a hypercomplex extreme learning machine (HELM) from a different perspective for the classification of multisource information. A hypercomplex representation [22, 23] is introduced in the ELM theory. Multisource features are employed to construct the hypercomplex space, and hypercomplex operation rules are applied to determine the output weights of SLFNs. In addition, a fusion strategy is performed on the hypercomplex output nodes to obtain a decision.

As a typical kind of multisource information processing problem, multispectral palmprint recognition has gained widespread attentions in recent years. Some previous works tried to process the multisource information using a fusion operation. For example, Lu et al. [24] completed an illumination-invariant palmprint recognition system by fusing the multispectral images at image level, in which a FABEMD+WFC fusion framework was developed. Similarly, Xu et al. [25] fused the multispectral images using a digital shearlet transform based method and then classified the fused images with the extreme learning machine. Gumaei et al. [26] proposed a kind of Gabor-based feature extraction method and employed the optimal spectral band to determine the identities. The same authors [27] further utilized a hybrid feature extraction method named HOG-SGF instead of Gabor-based one to represent the multispectral palmprint images. Recently, Gumaei et al. [28] developed a new anti-spoof multispectral biometric cloud-based identification approach for privacy and security of cloud computing, in which a tree-complex wavelet transform was applied to complete the multispectral fusion task and Gabor features were used to represent the fusion images. Different from a fusion view, in this paper we try to address the multispectral palmprint recognition problem by the proposed HELM framework, in which the fusion stage could be circumvented. To evaluate the performance of the proposed method, we conduct some experiments using the PolyU and CASIA multispectral palmprint databases [29–33]. Palmprint images from multispectral bands are employed to construct the hypercomplex representation.

The remainder of this paper is organized as follows: Section 2 provides a brief review of the ELM, describes the HELM theory and introduces the application of HELM in multispectral palmprint recognition. Section 3 illustrates the experimental results of the proposed method, which is tested using the PolyU and CASIA multispectral palmprint databases. Some concluding remarks are provided in the last section.

## 2 Related work

### 2.1 Extreme learning machine

The ELM is a novel learning method for SLFNs that randomly assigns the hidden layer and analytically determines the output weights of SLFNs. For *N* distinct training data {**x**_*i*_,**t**_*i*_},*i* = 1,2,⋯,*N*, **x**_*i*_ is a 1×*n* input vector, and **t**_*i*_ is a 1×*m* output vector with only one entry (correspond to the class to which **x**_*i*_ belongs) equal to one. *n* is the dimension of the input data, and *m* is the number of classes. To train an SLFN with $\tilde{N}$ hidden nodes, the appropriate input weight vectors $\alpha_{j},\;j=1,2,\cdots ,\tilde{N}$ and output weight vectors $\beta_{j},\;j=1,2,\cdots ,\tilde{N}$ are required, such that

$$f_{\tilde{N}}(x_{i})=\sum_{j=1}^{\tilde{N}}g(x_{i}^{e}\alpha_{j})\beta_{j}=t_{i},\;i=1,2,\cdots ,N,$$ (1)

where **α**_*j*_ is a (*n*+1)×1 vector that connects the input nodes to the *jth* hidden node, **β**_*j*_ is a 1×*m* vector that connects the *jth* hidden node to the output nodes, $x_{i}^{e}$ is the augmenting vector of **x**_*i*_ with the format of [**x**_*i*_,1]∈*R*^*n*+1^, and *g*(*x*) is the activation function.

This formula can be compactly written as

Hβ=T, (2)

where $H={\left[\begin{array}{ccc} g(x_{1}^{e}\alpha_{1}) & \cdots & g(x_{1}^{e}\alpha_{\tilde{N}})\\ \vdots & \cdots & \vdots \\ g(x_{N}^{e}\alpha_{1}) & \cdots & g(x_{N}^{e}\alpha_{\tilde{N}}) \end{array}\right]}_{N\times \tilde{N}}$, $\beta ={\left[\begin{array}{c} \beta_{1}\\ \vdots \\ \beta_{\tilde{N}} \end{array}\right]}_{\tilde{N}\times m}$ and $T={\left[\begin{array}{c} t_{1}\\ \vdots \\ t_{N} \end{array}\right]}_{N\times m}$. The hidden layer output matrix **H** is compactly described as

$$H=g(x^{e}\alpha ),$$ (3)

where $x^{e}={\left[\begin{array}{c} x_{1}^{e}\\ \vdots \\ x_{N}^{e} \end{array}\right]}_{N\times (n+1)}$ and $\alpha ={\left[\alpha_{1}\;\cdots \;\alpha_{\tilde{N}}\right]}_{(n+1)\times \tilde{N}}$.

Generally, a typical ELM training process consists of two main steps. The first step is to calculate the hidden layer output matrix with the random map **α** and a nonlinear piecewise continuous function, such as the following sigmoid function, sin function and atan function:

1. Sigmoid function:
  $$H(i,j)=\frac{1}{1+\exp(-x_{i}^{e}\alpha_{j})}$$ (4)
2. Sin function:
  $$H(i,j)=\sin(x_{i}^{e}\alpha_{j})$$ (5)
3. Atan function:
  $$H(i,j)=atan(x_{i}^{e}\alpha_{j})$$ (6)
  where **H**(*i*,*j*) is the value of **H** at the position (*i*, *j*).

A remarkable characteristic of ELM is that the input weight matrix **α** of the hidden nodes can be randomly generated according to any continuous probability distribution, for example, the uniform distribution on [–1,1]. ELM distinctly differs from conventional feedforward neural networks. As demonstrated by Eq (2), the only parameters that need to be optimized in the training process are the output weights $\beta ={\left[\begin{array}{c} \beta_{1}\\ \vdots \\ \beta_{\tilde{N}} \end{array}\right]}_{\tilde{N}\times m}$ between the hidden nodes and the output nodes. Mathematically, training an SLFN by an ELM can be transformed into solving a regularized least squares problem, as illustrated in Eq (2). Additional iterative steps are not required to tune the parameters of SLFNs, which are significantly more efficient than BP-like algorithms.

In the second step, ELM attempts to determine the output weights by minimizing the following loss function:

$$\begin{array}{l} E=\underset{\beta_{1},\beta_{2},\cdots ,\beta_{\tilde{N}}}{\min}\sum_{j=1}^{\tilde{N}}(g(x_{i}^{e}\alpha_{j})\beta_{j}-t_{i}{)}^{2}\\ \;=\underset{\beta \in R^{\tilde{N}\times m}}{\min}\left\|g(x^{e}\alpha )\beta -T\right\|\\ \;=\underset{\beta \in R^{\tilde{N}\times m}}{\min}\left\|H\beta -T\right\| \end{array}.$$ (7)

Huang et al. [4] proved that if the activation function *g* is infinitely differentiable, for $N=\tilde{N}$ arbitrary distinct samples {**x**_*i*_,**t**_*i*_},*i* = 1,2,⋯,*N*, for any randomly assigned **α** according to any continuous probability distribution, the hidden layer output matrix **H** is invertible and ‖**Hβ**−**T**‖ = 0. Thus, the output weight matrix **β** can be calculated by

$$\beta =H^{-1}T,$$ (8)

where **H**^−1^ denotes the inverse matrix of **H**.

In most cases, the number $\tilde{N}$ of hidden nodes is significantly less than the number *N* of distinct training samples, **H** is a non-square matrix, and an inverse matrix for **H** does not exist. Huang has provided another method for finding the smallest norm least squares solution of Eq (2), that is,

$$\beta =(H^{T}H+\frac{1}{C}I{)}^{-1}H^{T}T,$$ (9)

where **H**^*T*^ is the transpose of **H**, *C* is a penalty coefficient, and **I** is an identity matrix with the size $\tilde{N}\times \tilde{N}$.

Here, the procedures for training an SLFN using ELM theory are as follows:

Step 1: Initialize the number $\tilde{N}$ of hidden nodes; note that $\tilde{N}\leq N$.
Step 2: Select the suitable activation function *g*.
Step 3: Randomly assign the input weight matrix **α**.
Step 4: Construct the output matrix **H** of the hidden layer.
Step 5: Calculate the output weight matrix **β**.

### 2.2 Hypercomplex extreme learning machine

To classify the multisource patterns, an invariant of ELM—Hypercomplex extreme learning machine—is presented. Instead of using a fusion strategy to combine the multisource information, HELM is built on hypercomplex representation, by which the model converts the multisource features into a hypercomplex space. It circumvents the process of designing fusion rules and therefore avoids the interference due to someone’s limited knowledge. In addition, HELM takes advantages of all multispectral images and learns the model parameters adaptively according to training data. Thus HELM could be more efficient and accurate than the fusion-based strategy. To elaborate on the HELM model, initially we must introduce some basic concepts of hypercomplex operation. Mathematically, a hypercomplex number is a linear combination of a real scalar and the fixed number *d* of imaginary units:

$$y=y^{\left(1\right)}+y^{\left(2\right)}e_{1}+y^{\left(3\right)}e_{2}+\cdots +y^{\left(d+1\right)}e_{d},$$ (10)

where *y*^(1)^,*y*^(2)^,⋯,*y*^(*d*+1)^ are real numbers, and *e*_1_,*e*_2_,⋯,*e*_*d*_ are the imaginary units. They have the following relationship:

$$e_{1}{}^{2}=e_{2}{}^{2}=\cdots =e_{d}{}^{2}=e_{1}e_{2}\cdots e_{d}=-1,$$ (11)

y^*^ denotes the conjugate of *y* and is calculated by

$$y^{*}=y^{\left(1\right)}-y^{\left(2\right)}e_{1}-y^{\left(3\right)}e_{2}-\cdots -y^{\left(d+1\right)}e_{d}.$$ (12)

The norm of a hypercomplex number is defined as

$$\left|y\right|=\sqrt{yy^{*}}=\sqrt{{\left(y^{\left(1\right)}\right)}^{2}+{\left(y^{\left(2\right)}\right)}^{2}+\cdots +{\left(y^{\left(d+1\right)}\right)}^{2}}.$$ (13)

**Fig 1:**
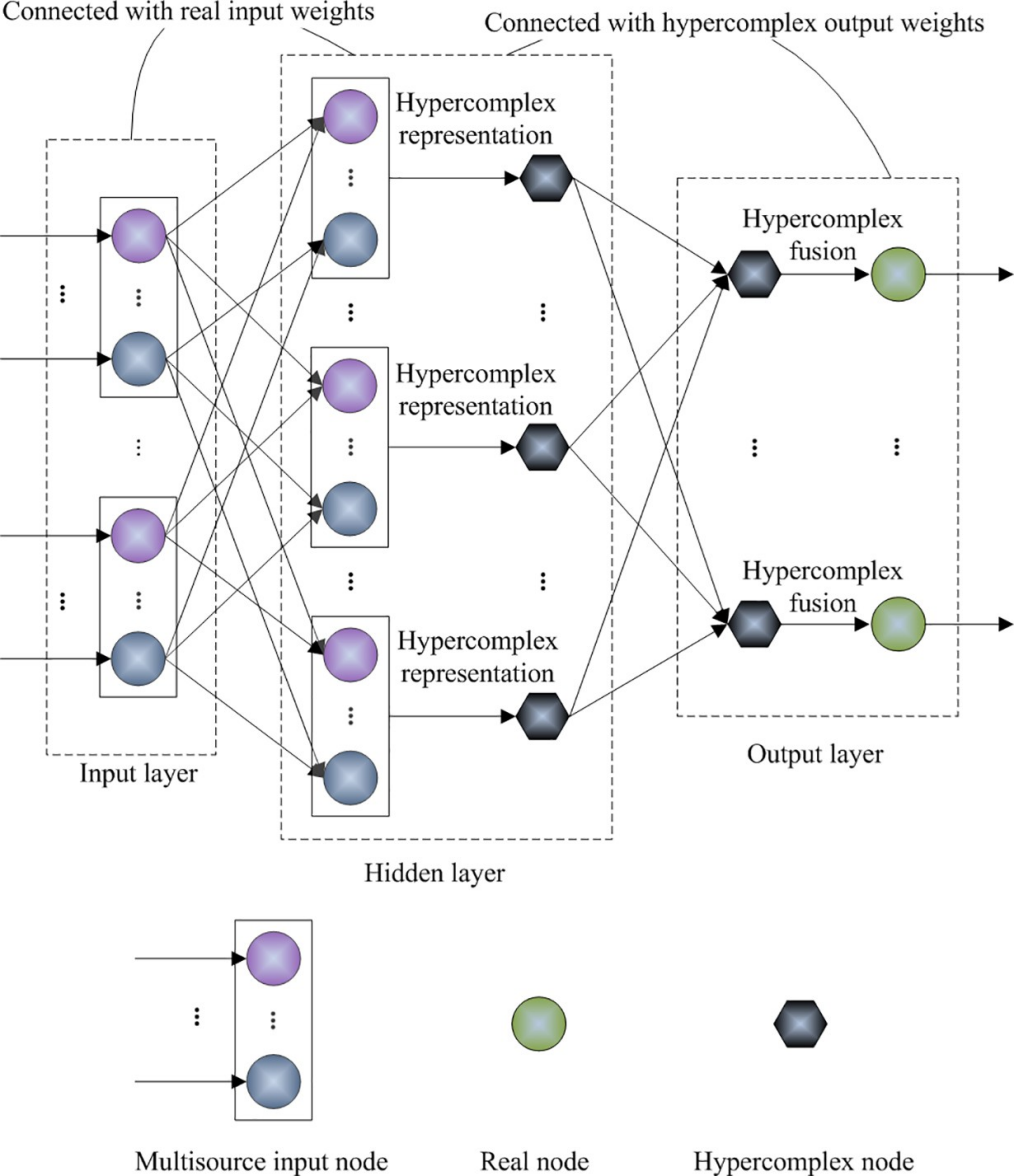
Structure of the proposed HELM model.

HELM aims to extend the extreme learning theory to hypercomplex space. In the case of classification of multisource features, HELM utilizes each type of feature to construct a hypercomplex matrix. Then, the weights of an SLFN are analytically determined with the hypercomplex operation rules. Fig 1 shows the structure of the proposed HELM network, which primarily consists of four key stages: mapping the multisource features into the hidden layer using the randomly generated real input weights, constructing the hypercomplex hidden layer output matrix, calculating the hypercomplex output weight matrix, and performing a fusion strategy on the output nodes to achieve a final decision.

For *N* distinct training samples with multisource features, i.e., {**x**_*i*_^(1)^,**x**_*i*_^(2)^,**x**_*i*_^(3)^,⋯,**x**_*i*_^(*d*+1)^,**t**_*i*_}, *i* = 1,2,⋯,*N*, where **x**_*i*_^(*j*)^∈*R*^1×*n*^ denotes the *jth* attribute of sample *i*, the core task for HELM is to determine the input weights and output weights of an SLFN. Similar to the settings in the ELM, we take a randomly generated map as the input weights. Each attribute of the training samples is mapped into the hidden layer as

$$H^{\left(j\right)}=g(x^{e(j)}\alpha^{\left(j\right)}),\;j=1,2,\cdots ,d+1,$$ (14)

where $x^{e(j)}={\left[\begin{array}{c} x_{1}^{e(j)}\\ \vdots \\ x_{N}^{e(j)} \end{array}\right]}_{{}_{N\times (n+1)}}$ and $\alpha^{\left(j\right)}={\left[\alpha_{1}{}^{\left(j\right)}\;\cdots \;\alpha_{\tilde{N}}{}^{\left(j\right)}\right]}_{(n+1)\times \tilde{N}}$. **H**^(*j*)^ denotes the hidden layer output matrix for the *jth* attribute, and **x**_*i*_^*e*(*j*)^ is the augmenting vector of **x**_*i*_^(*j*)^ with the format of [**x**_*i*_^(*j*)^ 1]∈*R*^*n*+1^. **α**^(*j*)^ is the real input weight matrix for the *jth* attribute. The input layer and the hidden layer are connected with the set of real input weight matrices **α**^(*j*)^, *j* = 1,2,⋯,*d*+1.

A hypercomplex output matrix 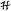 of the hidden layer is constructed using a hypercomplex representation, that is,

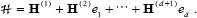
(15)

For sample *i*, different attributes share the same output vector **t**_*i*_. Thus, the hypercomplex output vector can be constructed as

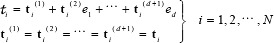
(16)

This vector can be compactly described as

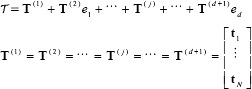
(17)
where **T**^(*j*)^ denotes the output matrix for the *jth* attribute, and 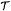 is the hypercomplex output matrix of an SLFN.

Having obtained 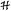 and 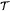, we can solve the hypercomplex output weight matrix 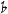 according to the following equations:

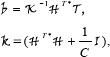
(18)
where 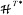 is the hypercomplex transposition-conjugate matrix of 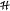. 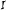 is the hypercomplex identity matrix with the size of $\tilde{N}\times \tilde{N}$. 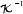 is the hypercomplex matrix inversion and is calculated by a blockwise recursion process described as

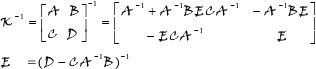
(19)
where 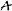, 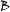, 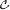 and 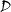 are the hypercomplex matrix sub-blocks of arbitrary size. To be inverted, 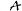 must be square. If the number of the rows (or columns) of 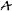 exceeds one, 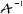 can be recursively solved by Eq (19). When 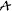 is scalar, 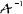 is computed by

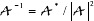
(20)
where 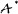 is the conjugate of 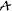, and 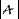 is the norm of 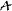. The same procedures are performed in the calculation of 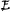.

With Eq (18), the output weight matrix of the hidden layer is obtained. The hidden layer and the output layer are connected with hypercomplex weights. The input weights **α**^(*j*)^, *j* = 1,2,⋯,*d*+1 and the output weights 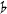 of an SLFN are determined. The input weights are a series of real matrices, and the output weights are represented using a hypercomplex matrix.

Once the training process is completed, a sum rule-based fusion strategy is performed on the hypercomplex output nodes, which considers the information from multisource features. Let 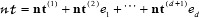 denote the hypercomplex output of a HELM network for a new sample with multisource features {**nx**^(1)^,**nx**^(2)^,⋯,**nx**^(*d*+1)^}. The final fusion result can be achieved by

$$f(j)=\sum_{k=1}^{d+1}\left\{\frac{nt^{\left(k\right)}(j)-\min\_t^{\left(k\right)}(j)}{\max\_t^{\left(k\right)}(j)-\min\_t^{\left(k\right)}(j)}\right\}$$ (21)

where **f**(*j*) denotes the *jth* element of the fusion result **f**∈*R*^1×*m*^. **nt**^(*k*)^(*j*) denotes the *jth* element of **nt**^(*k*)^. min_**t**^(*k*)^(*j*) and max_**t**^(*k*)^(*j*) are calculated by

$$\min\_t^{\left(k\right)}(j)=\min\left\{t_{1}{}^{\left(k\right)}(j),t_{2}{}^{\left(k\right)}(j),\cdots ,t_{N}{}^{\left(k\right)}(j)\right\}$$ (22)

$$\max\_t^{\left(k\right)}(j)=\max\left\{t_{1}{}^{\left(k\right)}(j),t_{2}{}^{\left(k\right)}(j),\cdots ,t_{N}{}^{\left(k\right)}(j)\right\}$$ (23)

where **t**_*i*_^(*k*)^(*j*) is the *jth* element of the **t**_*i*_^(*k*)^. **t**_*i*_^(*k*)^ is the HELM network output of the training sample *i* for the *kth* attribute.

Here, the procedures for training and testing an SLFN using HELM theory are as follows:

Step 1: Initialize the number $\tilde{N}$ of hidden nodes; note that $\tilde{N}\leq N$.
Step 2: Select the suitable activation function *g*.
Step 3: Randomly assign the input weight matrix **α**^(*j*)^, *j* = 1,2,⋯,*d*+1.
Step 4: Construct the hypercomplex output matrix H of the hidden layer.
Step 5: Calculate the hypercomplex output weight matrix 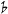.
Step 6: Obtain the fusion result using a sum rule.

### 2.3 Multispectral palmprint recognition using HELM

**Fig 2:**
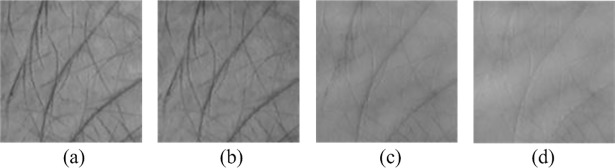
**Multispectral palmprint sample:** (a) Blue, (b) Green, (c) Red and (d) Near-infrared.

To evaluate the performance of the proposed HELM network, we have applied it to multispectral palmprint recognition. Images captured from different spectral bands are taken as the multisource features. Fig 2 demonstrates a multispectral palmprint sample.

**Fig 3:**
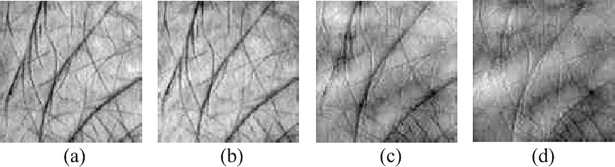
**Multispectral palmprint sample after intensity normalization:** (a) Blue, (b) Green, (c) Red and (d) Near-infrared.

Before using the HELM network to classify the multispectral palmprint images, the intensity normalization process illustrated in Eq (24), must be implemented on the palmprint images to remove the global intensity influence.

$$I(x,y)=\frac{I(x,y)-\min\_v}{\max\_v-\min\_v}$$ (24)

where **I**(*x*,*y*) denotes the pixel value of image **I** at position (*x*,*y*). min_*v* and max_*v* are the minimum and maximum, respectively, of all pixels in **I**. Fig 3 shows the palmprint images after intensity normalization operation.

**Fig 4:**
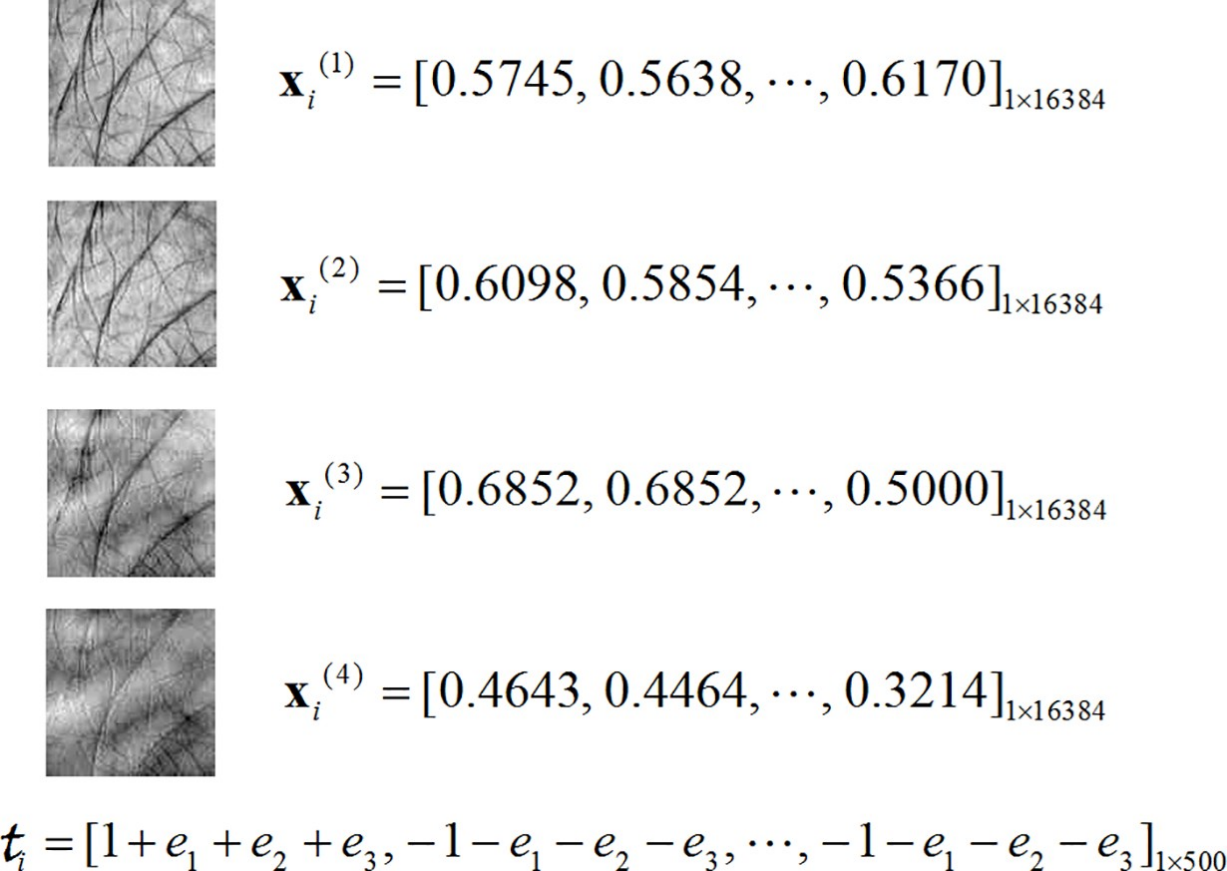
Typical input for HELM network.

As the input features of HELM network, an image must be adjusted to a row vector. Fig 4 lists typical training data for the HELM network. **x**_*i*_^(1)^, **x**_*i*_^(2)^, **x**_*i*_^(3)^ and **x**_*i*_^(4)^ are the multisource input features, i.e., the multispectral images captured at Blue, Green, Red and Near-infrared (NIR) bands. t_*i*_ denotes the hypercomplex output of the HELM network. The entry with the value of 1+*e*_1_+*e*_2_+*e*_3_ denotes the class to which sample *i* belongs.

With all training data, we can follow the steps described in HELM theory to train an SLFN and complete the palmprint recognition task.

## 3 Experimental results and performance analysis

In this section, we present the experimental results and assess the performance of the proposed HELM method. All experiments have been conducted on a computer with a 2.50 GHz Intel core processor and 8 GB memory. MATLAB 2017a was utilized as the simulation software.

### 3.1 Database description and evaluation criteria

To demonstrate the effectiveness of the proposed method, we conducted a series of experiments using the following two public multispectral palmprint databases.

**Fig 5:**
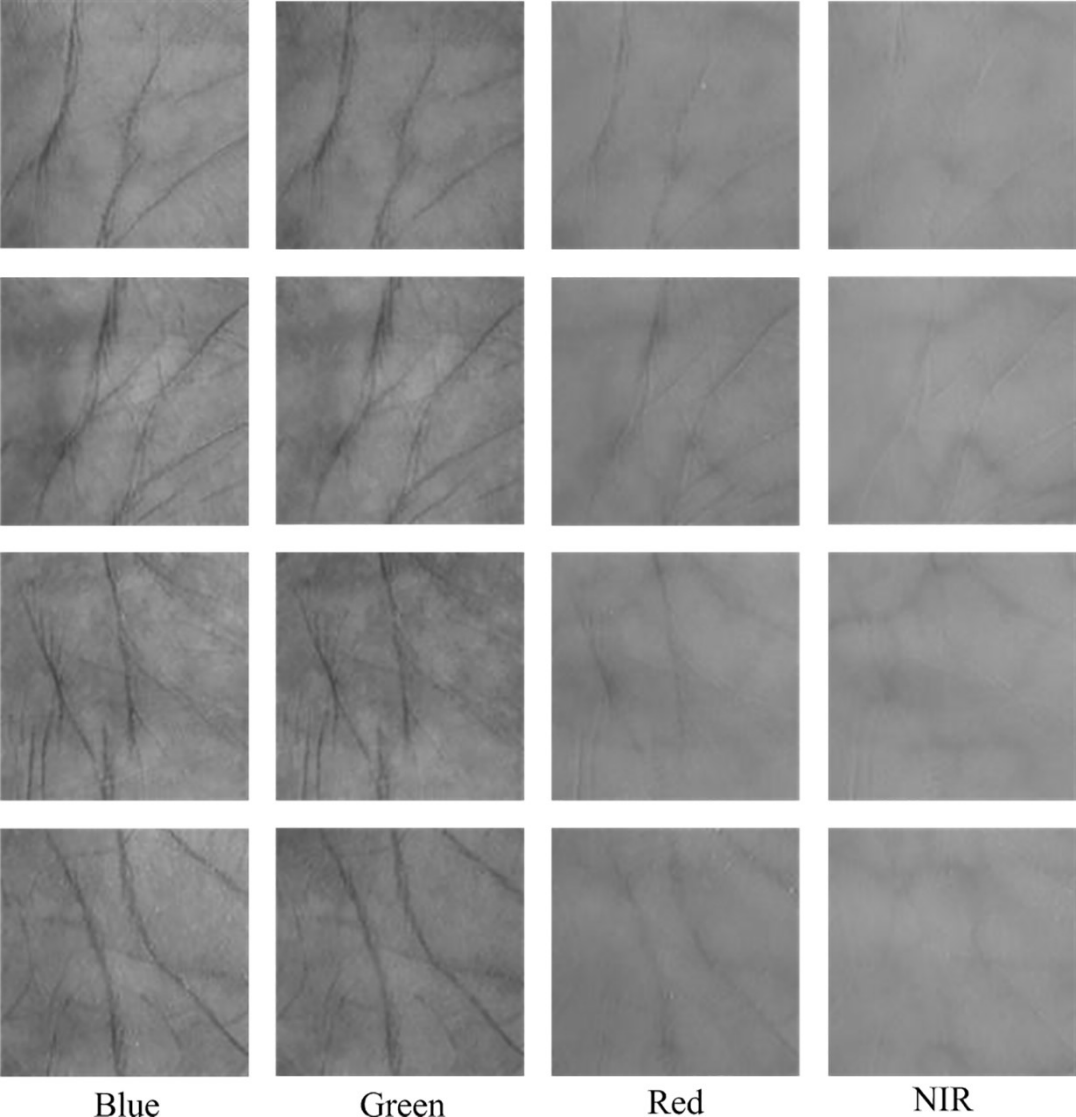
Multispectral palmprint samples in the PolyU database.

The PolyU database is [29–32] created by Hong Kong Polytechnic University. The database consists of 24000 plamprint images collected from 250 volunteers, who comprised 195 males and 55 females. The age of each volunteer ranged from 20 to 60 years old. During the acquisition process, each volunteer was sampled 12 times in two separate sessions for his/her left and right palms. The palmprint images were acquired at four spectral bands, i.e., Red, Green, Blue and NIR. For the convenience of researchers, the Hong Kong Polytechnic University provides the region of interest (ROI) images with the size 128×128. Fig 5 shows some multispectral palmprint samples in the PolyU database.

**Fig 6:**
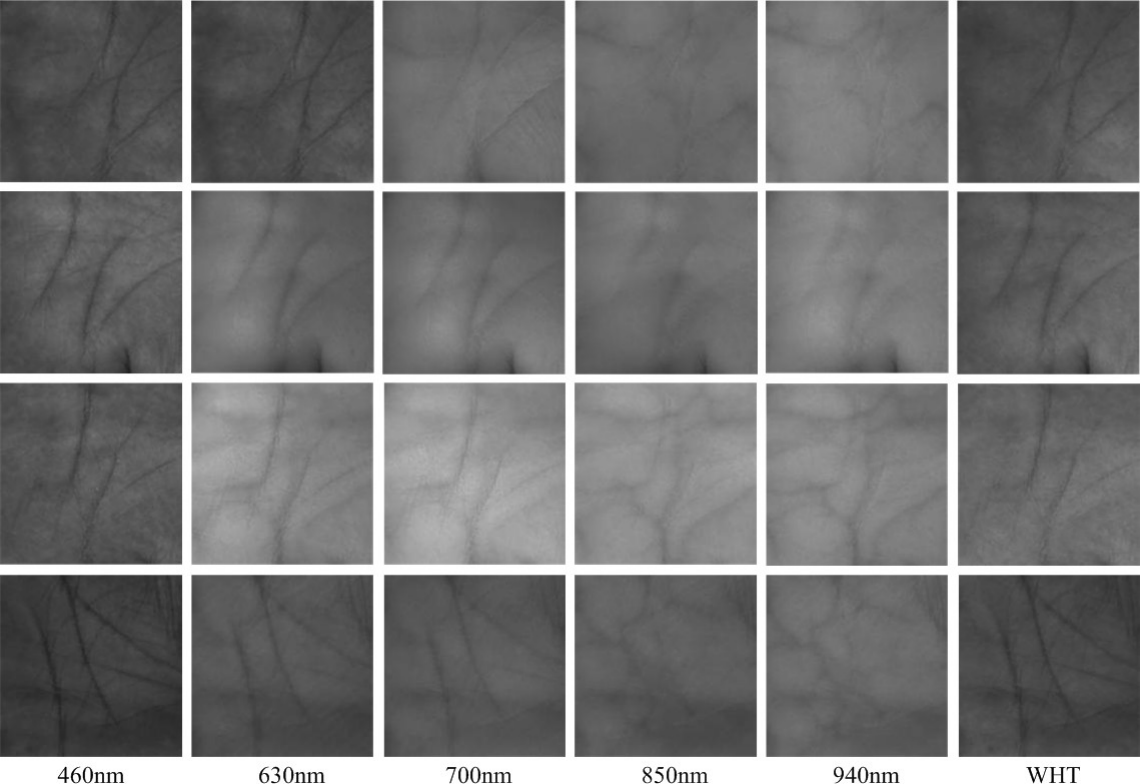
Multispectral palmprint samples in the CASIA database.

The CASIA database [33] is provided by the Chinese Academy of Sciences’ Institute of Automation. It has 7200 palmprint images in total collected from 100 volunteers. The acquisition was performed in two separate sessions with a minimum time interval of one month. In one session, each volunteer was required to provide 3 samples for his/her left and right palm respectively. Each sample was captured at 460nm, 630nm, 700nm, 850nm, 940nm and white light (WHT) spectral bands respectively. Fig 6 shows some multispectral palmprint images in the CASIA database.

The performance of the proposed method is evaluated in terms of recognition accuracy and computational cost. In the recognition process, a certain number of multispectral palmprint images are treated as the testing samples. If the determined class label of one testing sample is the same with its actual label, it is considered as a correctly recognized sample. Otherwise, it is an incorrectly recognized one. Then the recognition accuracy is defined as

$$RA=N_{c}/N$$ (25)

where *N*_*c*_ is the number of the correctly recognized samples in the testing group. *N* is the number of the samples in the testing group.

The computational cost including the training and testing time is also used to compare the performance of the method. The training time is referred to as the time cost for constructing the HELM model using the training data. And the testing time is the time cost for determining the class labels of the testing samples using the trained HELM model.

### 3.2 Result analysis of the proposed method

**Fig 7:**
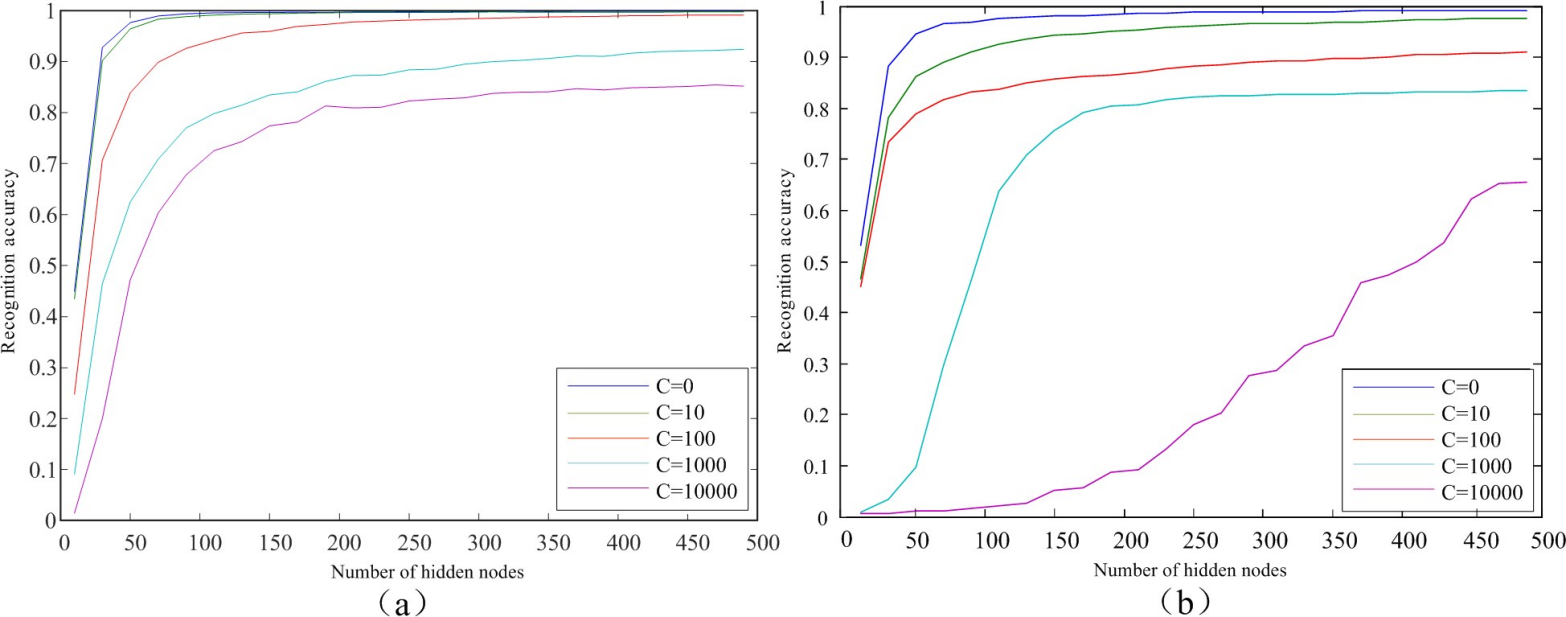
**Performance with different settings in HELM:** (a) PolyU, (b) CASIA.

To complete the training of the HELM network and achieve excellent generalization performance, the penalty coefficient *C* and the number $\tilde{N}$ of hidden nodes need to be appropriately chosen. We have employed different settings of *C* and $\tilde{N}$. The results of the experiments on the two databases are demonstrated in Fig 7. It is clear from the figure that no matter which database the experiments are conducted on, for a given penalty coefficient *C* the recognition accuracy has an increasing trend as the number $\tilde{N}$ of hidden nodes progressively increases. It converges to the optimal accuracy when the value of $\tilde{N}$ is sufficiently large. We observe that a smaller value of *C* yields a higher recognition accuracy. When *C* is zero, the best results are generated.

Considering the randomness of the HELM training method, ten repeated experiments were performed. We also tested the performance of the HELM network with different activation functions. The sigmoid, sin and atan functions were compared to determine which function can achieve the optimal result. Table 1 lists the recognition accuracies in the ten repetitions.

**Table 1:** Recognition accuracies of the ten repeated measurements with different activation functions.

Number of measurement	*RA* (%)					
	PolyU			CASIA		
	Sigmoid	Sin	Atan	Sigmoid	Sin	Atan
1	100	99.20	99.87	98.17	97.50	97.67
2	99.90	98.83	99.70	98.33	97.67	97.83
3	99.90	99.03	99.83	98.50	97.50	97.67
4	99.97	99.13	99.83	98.00	97.83	97.67
5	99.93	98.97	99.83	98.00	97.67	97.83
6	100	99.00	99.93	98.17	97.50	97.50
7	99.90	98.97	99.93	98.00	97.33	98.00
8	99.93	99.20	99.87	98.50	97.83	97.67
9	99.97	99.03	99.83	98.33	97.67	97.83
10	100	98.97	99.93	98.17	97.83	97.83

Based on these repeating results, Table 2 gives the statistical comparison by using the one-sided Two-sample Student *T*-test. It makes the hypothesis that two independent samples come from normal distributions with equal means and variances. The value of test statistic can be calculated as:

$$t=\frac{{\bar{X}}_{1}-{\bar{X}}_{2}}{\sqrt{\frac{(n_{1}-1)S_{1}^{2}+(n_{2}-1)S_{2}^{2}}{n_{1}-n_{2}-2}(\frac{1}{n_{1}}+\frac{1}{n_{2}})}}$$ (26)

where ${\bar{X}}_{1}$ and ${\bar{X}}_{2}$ denote the means of the two series of measurements, $S_{1}^{2}$ and $S_{2}^{2}$ denote the corresponding variances, *n*_1_ and *n*_2_ are the numbers of measurements in each series. The number of degrees of freedom for the Two-sample *T*-test is *n*_1_+*n*_2_−2. To complete this statistical comparison, the built-in Matlab function of “ttest2(x, y, α, ‘right’)” is used. Here, x and y denote the two series of measurements. α is the significance level. ‘right’ denotes the right-sided test.

**Table 2:** Student *T*-test of the ten repeated measurements with different activation functions.

Hypothesis	PolyU			CASIA		
	*t*	*p*	*T-*test	*t*	*p*	*T-*test
*H*_0_: *RA*_Sigmoid_≤*RA*_Sin_ *H*_1_: *RA*_Sigmoid_>*RA*_Sin_	23.59	2.740E-15	*H*_1_	7.158	5.746E-7	*H*_1_
*H*_0_: *RA*_Sigmoid_≤*RA*_Atan_ *H*_1_: *RA*_Sigmoid_>*RA*_Atan_	3.666	8.845E-4	*H*_1_	6.205	3.714E-6	*H*_1_
*H*_0_: *RA*_Atan_≤*RA*_Sin_ *H*_1_: *RA*_Atan_>*RA*_Sin_	19.30	8.902E-14	*H*_1_	1.670	0.0561	*H*_0_

In Table 2, *t* denotes the value of the test statistic, *p* denotes the probability of observing the given result if the null hypothesis *H*_0_ is true and *T*-test denotes the test result. The significance level α is set to be α = 0.0*5*. By making the comparison between the two activation functions of sigmoid and sin, it can be found that the *p* values for PolyU and CASIA databases are much less than the significance level α. Thus the null hypothesis *H*_0_ is rejected and the alternative hypothesis *H*_1_ is accepted, meaning that the sigmoid function can produce higher recognition accuracy than the sin function. Similarly, the inference that the sigmoid function outperforms the atan function can be obtained by making the *T*-test between the two functions of sigmoid and atan. As for the comparison between sin and atan functions, two different test results are achieved. The performance of the atan function is not significantly better than sin function for the CASIA database. It can be concluded that for the two databases, the sigmoid function consistently produces the highest recognition accuracy among the three types of activation functions. In the ten repeated measurements for the sigmoid function, the best recognition accuracies for the PolyU database and CASIA database are 100% and 98.50% respectively.

To evaluate the performance of the HELM network in the case of different combinations of input spectral bands, a series of experiments were conducted using different hypercomplex representations, i.e., *y* = *y*^(1)^+*y*^(2)^*e*_1_, *y* = *y*^(1)^+*y*^(2)^*e*_1_+*y*^(3)^*e*_2_, *y* = *y*^(1)^+*y*^(2)^*e*_1_+*y*^(3)^*e*_2_+*y*^(4)^*e*_3_ or *y* = *y*^(1)^+*y*^(2)^*e*_1_+*y*^(3)^*e*_2_+*y*^(4)^*e*_3_+*y*^(5)^*e*_4_. In the situation with a single input spectral band, the HELM degraded into a traditional ELM network. Considering the randomness in the training of the HELM network, ten repeated runs were performed. The average accuracy and the corresponding standard deviation were employed as the assessment. We also applied three activation functions in the network. Tables 3 and 4 illustrate the experimental results of different spectral combinations when testing the HELM method on the PolyU and CASIA multispectral databases. As shown in the Table 3, the recognition accuracies of the experiments with more than one input spectral band are higher than the recognition accuracies of the experiments with a single spectral band. The recognition results based on the HELM network are satisfied when the number of input spectral bands is two or three, which has proven that the proposed HELM network is applicable for any spectral band combination. We also observe that the sigmoid function achieves the optimal results among the three activation functions when the number of input spectral bands is two or three. Regarding the case of a single spectral band, the Atan function obtains the best results for the ELM model. Similarly, we can also obtain these conclusions from Table 4. For the CASIA database, using the proposed HELM model with multispectral palmprint images can obviously improve the performance of palmprint recognition. The sigmoid activation function provides the optimal recognition accuracies for these experiments with more than one input spectral band.

**Table 3:** Recognition accuracies for different combinations of spectral bands on the PolyU database.

Input spectral bands	*RA* (%)		
	Sigmoid	Sin	Atan
Blue	94.79±0.18	93.64±0.14	97.13±0.16
Green	93.55±0.19	92.23±0.16	96.54±0.19
Red	94.28±0.20	92.67±0.13	96.68±0.13
NIR	94.65±0.15	91.98±0.19	97.03±0.19
Blue Green	98.63±0.14	97.24±0.12	98.37±0.13
Blue Red	99.42±0.14	98.35±0.11	99.31±0.19
Blue NIR	99.66±0.10	98.94±0.12	99.58±0.10
Green Red	99.31±0.16	98.58±0.13	99.15±0.15
Green NIR	99.74±0.10	98.62±0.14	99.44±0.12
Red NIR	99.25±0.13	97.76±0.15	99.03±0.10
Blue Green Red	99.68±0.08	98.59±0.11	99.41±0.13
Blue Green NIR	99.87±0.07	99.27±0.09	99.76±0.08
Green Red NIR	99.86±0.04	98.70±0.07	99.76±0.05

**Table 4:** Recognition accuracies for different combinations of spectral bands on the CASIA database.

Input spectral bands	*RA* (%)		
	Sigmoid	Sin	Atan
460nm	90.55±0.46	90.38±0.51	91.17±0.30
630nm	91.85±0.48	91.81±0.36	92.13±0.33
700nm	90.53±0.42	90.37±0.49	90.87±0.30
850nm	92.25±0.35	92.07±0.48	92.36±0.28
940nm	93.23±0.42	93.15±0.37	93.50±0.22
WHT	92.50±0.37	92.22±0.38	92.75±0.21
460nm 940nm	96.86±0.17	96.38±0.11	96.53±0.21
940nm WHT	95.40±0.16	95.11±0.25	94.93±0.22
460nm 850nm WHT	97.12±0.24	96.95±0.24	96.97±0.22
460nm 940nm WHT	97.58±0.23	97.52±0.12	97.55±0.21
460nm 700nm 850nm 940nm	97.88±0.14	97.33±0.21	97.38±0.22
460nm 850nm 940nm WHT	97.93±0.16	97.68±0.16	97.73±0.22
460nm 630nm 700nm 850nm 940nm	97.90±0.11	97.30±0.11	97.68±0.17
460nm 630nm 850nm 940nm WHT	98.03±0.12	97.89±0.14	97.93±0.15

HELM employs a hypercomplex representation to complete the classification task of multisource features. To verify its effectiveness, a comparison was performed with two different strategies for ELM to process the multisource features, i.e., fusing the multisource features either at a feature level or a matching score level. These methods were compared in terms of computational cost and recognition accuracy. Similarly, ten repeated runs were performed. The time and accuracy were employed in the assessment. As reported in Table 5, we conclude that for either benchmark database, the hypercomplex representation-based method obtains the highest recognition accuracy and maintains a distinct advantage over the other two strategies. Regarding computation time, although the hypercomplex representation based-method cannot compete with the feature level fusion based method in terms of testing time, it requires the lowest training time and provides the highest recognition accuracy. The hypercomplex representation strategy outperforms both of the comparison methods.

**Table 5:** Comparison with different strategies of processing the multisource features of ELM.

Method	PolyU			CASIA		
	Training Time (s)	Testing Time (s)	*RA* (%)	Training Time (s)	Testing Time (s)	*RA* (%)
Feature level fusion	3.95±0.11	0.65±0.09	97.13±0.14	2.14±0.11	0.21±0.04	95.40±0.17
Matching score level fusion	7.52±0.21	1.63±0.08	92.74±0.12	3.26±0.12	0.31±0.08	90.56±0.45
Hypercomplex representation	3.42±0.15	1.68±0.09	99.95±0.04	1.34±0.11	0.19±0.05	98.22±0.19

**Fig 8:**
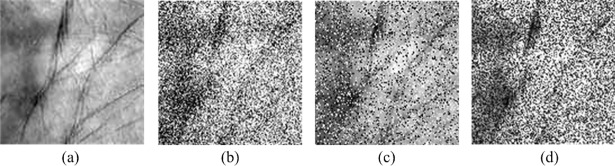
**Pure and noisy palmprint samples:** (a) Pure image, (b) Image with Gaussian white noise, (c) Image with Salt & Pepper noise, (d) Image with Speckle noise.

A comparison was made with some state-of-art multispectral palmprint recognition methods, including two image level fusion methods, two matching score level fusion methods, a QPCA+QDWT method and two improved ELM-based methods. In addition, we also investigated the performance of the HELM model when using different features as the input. A dimensionality reduction method and a texture feature extraction method—PCA [34] and LBP [35]—were employed to extract the palmprint features. The experiments were conducted on the pure PolyU and CASIA databases as well as the corresponding manually generated ones by introducing different kinds of noises. Fig 8 demonstrates the manually generated palmprint samples used in these experiments. The Gaussian white noise with mean 0 and standard deviation 36, the Salt & Pepper noise with 10% noise density and the Speckle noise with variance 0.05 were utilized respectively to generate the noisy palmprint images. Table 6 lists the recognition accuracies of the comparison methods. We can discover that the HELM-based and PCA+HELM-based multispectral palmprint recognition methods consistently outperform the fusion-related methods and the QPCA+QDWT method on the testing databases. Although the two improved ELM-based methods could achieve quite satisfactory results on the PolyU database, the performance degrades when they are tested on the CASIA database. The LBP+HELM method could produce the highest recognition accuracies on the pure PolyU and CASIA databases (100% and 99.83). However, the recognition accuracies decrease seriously when the images are corrupted with noises. This is because the LBP features depend on the local structure of images and are very sensitive to the variation of pixel value.

**Table 6:** Recognition accuracy comparison with different multispectral palmprint recognition methods.

Method	*RA* (%)							
	PolyU				CASIA			
	Pure	Gaussian	Salt & Pepper	Speckle	Pure	Gaussian	Salt & Pepper	Speckle
Image level fusion by PCA [36]	95.53	94.03	94.40	94.23	93.67	92.33	93.33	92.17
Matching score level fusion by PCA [37]	97.97	96.83	97.07	96.67	96.50	96.16	96.33	95.83
Image level fusion by DWT [32]	96.60	71.20	61.07	71.87	94.00	65.67	58.50	65.33
Matching score level fusion by DWT [38]	98.00	97.46	97.37	96.97	96.67	96.16	95.83	95.50
QPCA+QDWT [39]	98.83	98.60	97.90	97.90	96.67	96.33	96.17	95.67
MPELM [25]	99.53	98.80	98.87	98.63	90.00	84.67	88.00	85.00
TELM [24]	99.95	99.00	99.17	99.10	96.83	94.83	96.00	93.50
HELM	100	99.80	99.70	99.67	98.50	98.00	97.83	97.67
PCA+HELM	100	99.93	99.83	99.90	98.50	98.17	98.00	97.83
LBP+HELM	100	93.70	91.90	90.43	99.83	93.17	90.83	89.50

Table 7 gives the statistical comparison of the ten methods in Table 6 by using the one-sided Two-sample Student *T*-test. The significance level is set to be α = 0.0*5*. Here, the meanings of *t*, *p* and *T*-test are same with those in Table 2 and the value of *t* is calculated as shown in Eq (26). IPCA denotes the method of “Image level fusion by PCA”. MPCA denotes the method of “Matching score level fusion by PCA”. IDWT denotes the method of “Image level fusion by DWT”. MDWT denotes the method of “Matching score level fusion by DWT”. QPCA denotes the method of “QPCA+QDWT”. By making the comparison between HELM method (Or PCA+HELM method) with the fusion-related methods, the QPCA+QDWT method and the improved ELM-based methods, we can find that the values of *p* are obviously less than the significance level α. Therefore the alternative hypothesis *H*_1_ is accepted. That is to say, the HELM method (Or PCA+HELM method) could achieve higher recognition accuracies than the comparison methods from a statistical viewpoint. In addition, it is observed that the LBP+HELM method is not significantly better than the comparison methods due to the noise effect. As for the Student *T*-test between the three HELM-related methods, the test results show that the PCA+HELM method can produce the highest recognition accuracies.

**Table 7:** Student *T*-test between different multispectral palmprint recognition methods.

Hypothesis		*t*	*p*	*T-*test
*H*_0_: *RA*_HELM_≤*RA*_IPCA_	*H*_1_: *RA*_HELM_>*RA*_IPCA_	9.867	5.512E-8	*H*_1_
*H*_0_: *RA*_HELM_≤*RA*_MPCA_	*H*_1_: *RA*_HELM_>*RA*_MPCA_	5.304	5.570E-5	*H*_1_
*H*_0_: *RA*_HELM_≤*RA*_IDWT_	*H*_1_: *RA*_HELM_>*RA*_IDWT_	5.041	9.016E-5	*H*_1_
*H*_0_: *RA*_HELM_≤*RA*_MDWT_	*H*_1_: *RA*_HELM_>*RA*_MDWT_	4.620	1.986E-4	*H*_1_
*H*_0_: *RA*_HELM_≤*RA*_QPCA_	*H*_1_: *RA*_HELM_ >*RA*_QPCA_	2.982	4.952E-3	*H*_1_
*H*_*0*_:*RA*_HELM_≤*RA*_MPELM_	*H*_*1*_:*RA*_HELM_ >*RA*_MPELM_	2.506	1.259E-2	*H*_1_
*H*_*0*_:*RA*_HELM_≤*RA*_TELM_	*H*_*1*_:*RA*_HELM_ >*RA*_TELM_	1.764	4.974E-2	*H*_1_
*H*_0_: *RA*_PCA*+*HELM_≤*RA*_IPCA_	*H*_1_: *RA*_PCA*+*HELM_ >*RA*_IPCA_	10.17	3.777E-8	*H*_1_
*H*_0_: *RA*_PCA*+*HELM_≤*RA*_MPCA_	*H*_1_: *RA*_PCA*+*HELM_ >*RA*_MPCA_	5.660	2.944E-5	*H*_1_
*H*_0_: *RA*_PCA*+*HELM_≤*RA*_IDWT_	*H*_1_: *RA*_PCA*+*HELM_ >*RA*_IDWT_	5.065	8.618E-5	*H*_1_
*H*_0_: *RA*_PCA*+*HELM_≤*RA*_MDWT_	*H*_1_: *RA*_PCA*+*HELM_ >*RA*_MDWT_	4.929	1.110E-4	*H*_1_
*H*_0_: *RA*_PCA*+*HELM_≤*RA*_QPCA_	*H*_1_: *RA*_PCA+HELM_ >*RA*_QPCA_	3.227	3.040E-3	*H*_1_
*H*_*0*_:*RA*_PCA+HELM_≤*RA*_MPELM_	*H*_*1*_:*RA*_PCA+HELM_ >*RA*_MPELM_	2.559	1.137E-2	*H*_1_
*H*_*0*_:*RA*_PCA+HELM_≤*RA*_TELM_	*H*_*1*_:*RA*_PCA+HELM_ >*RA*_TELM_	1.905	3.874E-2	*H*_1_
*H*_0_: *RA*_LBP*+*HELM_≤*RA*_IPCA_	*H*_1_: *RA*_LBP*+*HELM_ >*RA*_IPCA_	-0.028	5.118E-1	*H*_0_
*H*_0_: *RA*_LBP*+*HELM_≤*RA*_MPCA_	*H*_1_: *RA*_LBP*+*HELM_ >*RA*_MPCA_	-2.047	9.700E-1	*H*_0_
*H*_0_: *RA*_LBP*+*HELM_≤*RA*_IDWT_	*H*_1_: *RA*_LBP*+*HELM_ >*RA*_IDWT_	3.880	8.336E-4	*H*_1_
*H*_0_: *RA*_LBP*+*HELM_≤*RA*_MDWT_	*H*_1_: *RA*_LBP*+*HELM_ >*RA*_MDWT_	-2.078	9.717E-1	*H*_0_
*H*_0_: *RA*_LBP*+*HELM_≤*RA*_QPCA_	*H*_1_: *RA*_LBP*+*HELM_ >*RA*_QPCA_	-2.380	9.840E-1	*H*_0_
*H*_*0*_:*RA*_LBP+HELM_≤*RA*_MPELM_	*H*_*1*_:*RA*_LBP+HELM_ >*RA*_MPELM_	0.265	3.974E-1	*H*_0_
*H*_*0*_:*RA*_LBP+HELM_≤*RA*_TELM_	*H*_*1*_:*RA*_LBP+HELM_ >*RA*_TELM_	-2.170	9.762E-1	*H*_0_
*H*_0_: *RA*_HELM_≤*RA*_PCA+HELM_	*H*_1_: *RA*_HELM_ >*RA*_PCA+HELM_	-2.516	5.975E-1	*H*_0_
*H*_0_: *RA*_PCA+HELM_≤*RA*_LBP+HELM_	*H*_1_: *RA*_PCA+HELM_ >*RA*_LBP+HELM_	3.595	1.464E-3	*H*_1_
*H*_0_: *RA*_HELM_≤*RA*_LBP+HELM_	*H*_1_: *RA*_HELM_ >*RA*_LBP+HELM_	3.509	1.737E-3	*H*_1_

## 4 Conclusions

In this paper, we have proposed HELM, which is a novel learning method for SLFNs. HELM introduces the hypercomplex representation concept into ELM theory. In contrast to the conventional ELM model, the proposed method maintains all merits of ELM, such as fast learning speed, excellent generalization ability and ease of implementation. HELM can easily complete the classification task of multisource features by benefitting from the hypercomplex representation. We have applied this method to the task of multispectral palmprint recognition to verify the actual performance. Comprehensive experiments carried out on the PolyU and CASIA multispectral palmprint databases have demonstrated that the proposed HELM network can obtain favorable results compared with several state-of-the-art multispectral palmprint recognition methods.
